# Influence of Microstructure and Mechanical Properties of Dissimilar Rotary Friction Welded Inconel to Stainless Steel Joints

**DOI:** 10.3390/ma16083049

**Published:** 2023-04-12

**Authors:** Akhil Reddy Beeravolu, Nagumothu Kishore Babu, Mahesh Kumar Talari, Ateekh Ur Rehman, Prakash Srirangam

**Affiliations:** 1 Department of Metallurgical and Materials Engineering, National Institute of Technology, Warangal 506004, India; bamm20205@student.nitw.ac.in (A.R.B.); talari@nitw.ac.in (M.K.T.); 2 Department of Industrial Engineering, College of Engineering, King Saud University, Riyadh 11451, Saudi Arabia; 3 Warwick Manufacturing Group, University of Warwick, Coventry CV4 7AL, UK; p.srirangam@warwick.ac.uk

**Keywords:** rotary friction welding, Inconel 718, AISI 316L, microstructure, hardness, mechanical properties

## Abstract

The present study aims to evaluate the microstructure, grain size, and mechanical properties of the dissimilar AISI 316L/Inconel 718 (IN 718) rotary friction welded joints under both the as-welded and post-weld heat treatment (PWHT) conditions. Because of reduced flow strength at elevated temperatures, the AISI 316L and IN 718 dissimilar weldments exhibited more flash formation on the AISI 316L side. At higher rotating speeds during friction welding, an intermixing zone was created at the weld joint interface due to the material softening and squeezing. The dissimilar welds exhibited distinctive regions, including the fully deformed zone (FDZ), heat-affected zone (HAZ), thermo-mechanically affected zone (TMAZ), and the base metal (BM), located on either side of the weld interface. The dissimilar friction welds, AISI 316L/IN 718 ST and AISI 316L/IN 718 STA, exhibited yield strength (YS) of 634 ± 9 MPa and 602 ± 3 MPa, ultimate tensile strength (UTS) of 728 ± 7 MPa and 697± 2 MPa, and % elongation (% El) of 14 ± 1.5 and 17 ± 0.9, respectively. Among the welded samples, PWHT samples exhibited high strength (YS = 730 ± 2 MPa, UTS = 828 ± 5 MPa, % El = 9 ± 1.2), and this may be attributed to the formation of precipitates. Dissimilar PWHT friction weld samples resulted in the highest hardness among all the conditions in the FDZ due to the formation of precipitates. On the AISI 316L side, prolonged exposure to high temperatures during PWHT resulted in grain growth and decreased hardness. During the tensile test at ambient temperature, both the as-welded and PWHT friction weld joints failed in the HAZ regions of the AISI 316L side.

## 1. Introduction

Nickel-based superalloy IN 718 finds primary applications in aerospace, gas turbines, combustors, turbochargers, rotors, and nuclear power plants due to its oxidation, corrosion, and creep resistance at high temperatures. Inconel 718 is one of the most widely used alloys in high-temperature applications. Inconel 718 alloy has considerably good weldability, owing to its resistance against strain age cracking [1,2,3,4]. Strain age cracking occurs due to the combination of residual stresses and the presence of small precipitates within the material. When the material is exposed to high temperatures for an extended period of time, these precipitates can grow, leading to the formation of cracks in the material. The good weldability characteristics of IN 718 are associated with the precipitation kinetics of the Ni_3_Nb, γ″ phase. Fusion welding is often used to fabricate Inconel 718 components, and during solidification, two types of eutectic components, NbC and Laves phases, are formed during the later stages of solidification [1,2,3,4]. The Laves phase forms at low temperatures and increases the tendency for hot/solidification cracking by extending the solidification temperature range [5,6,7,8]. IN 718 has a significant cost limitation; however, it can be utilized as a dissimilar joint with less expensive materials.

AISI 316L is a commonly used austenitic stainless steel recognized for its superior corrosion resistance, toughness, ductility, and formability. These low-cost structural materials are widely employed in various applications, including household items, cookware, cutlery, medical equipment, and aerospace and automotive structures, which are fabricated into different components [9]. Austenitic stainless steel is considered the most weldable among stainless steel and is commonly joined through fusion or resistance welding processes. However, these processes may cause sensitization in the heat-affected zone and promote hot cracking or solidification cracking. Sensitization occurs due to the formation and precipitation of chromium carbide at grain boundaries where carbon is predominantly found, leading to the depletion of chromium in the region and a subsequent decrease in corrosion resistance [9]. To prevent this problem, low-carbon stainless steel 316L can be used. During the fusion welding process, such as laser beam welding (LBW), gas tungsten arc welding (GTAW), metal inert gas welding (MIG), and electron beam welding (EBW), unexpected phase changes may occur, such as changes in delta ferrite formation, grain boundary corrosion, and sigma phase formation due to metallurgical changes at the weld interface.

The presence of delta ferrite in austenitic stainless steel can have several negative effects on its properties. Delta ferrite is a softer phase than austenite, which leads to a decrease in the material’s hardness and strength. It can also reduce the material’s ductility and toughness by acting as a preferential site for crack initiation and propagation. Additionally, delta ferrite can form at the grain boundaries and interdendritic regions, resulting in a decrease in the material’s corrosion resistance [10]. Lalam et al. [11] investigated the joining of two dissimilar metals, Inconel 718 and EN24, utilizing continuous drive friction welding. The welding parameters used included a frictional force of 28 kN, a rotational speed of 1422 rpm, an upset force of 41 kN, and a burn-off length of 5 mm. The researchers examined the microstructure and elemental diffusion across the joint interface and measured the joint’s tensile strength. The diffusion of elements, such as Cr, Ni, Nb, Mo, and Ti, from Inconel 718 to EN24, was observed after post-weld two-step aging treatment, while no change occurred after quench and tempering treatment, leading to intermetallic compound formation. The results showed that the dissimilar joint had a high tensile strength of 957 MPa after post-weld quenching and tempering treatment, with ductility of 7.5% when compared to the as-welded condition (tensile strength of 650 MPa and ductility of 15%). In contrast, the dissimilar welds subjected to post-weld two-step aging treatment exhibited a UTS of 650 MPa and ductility of 4%, which may be attributed to the precipitation of carbides. The study demonstrated that friction welding is an effective method for joining dissimilar metals and can produce high-strength joints with good ductility [11].

The joining of dissimilar metals has significantly increased due to the rapid development of new materials. The application areas of dissimilar metal joining include aerospace, nuclear power, automobile, marine, and chemical industries. Joining dissimilar metals is often more difficult than joining similar metals due to differences in the mechanical, physical, and metallurgical qualities of the parent metal. It is necessary to build high-quality joints between different metals for the specified applications mentioned above. In the friction welding process, a strong bond is formed at the interface between the materials to be joined via frictional heating and deformation. As the welding is performed in a solid state, there is no significant element migration. Friction welding is a solid-state welding process free from defects, such as segregation, porosity, solidification cracking, and liquation cracking, which are common in typical fusion welding processes [12].

Bansal et al. [13] studied the microwave welding of Inconel 718 and AISI 316L and reported that a dense joint was formed. EDAX analysis revealed the presence of Nb-enriched carbides in the weld zone, and in the tensile test, joint failure occurred in the weld zone due to the presence of laves phases/carbide particles. Hinojos et al. [14] explored joining AISI 316L and Inconel 718 using electron beam additive manufacturing technology. They concluded that welding features, such as the weld interface and HAZ, were substantially smaller than those formed via typical welding methods, such as GTAW, and they were comparable in size to those of EBW. In this method, the formation of Laves and NbC was observed to be lower when compared to conventional joining techniques. Ramkumar et al. [15] reported that Laves phases are the preferred sites for a stress-free crack generation. Microstructure analysis revealed that inclusions in the HAZ affected the tensile strength, resulting in brittle fracture. Sahu et al. [16] studied the influence of welding speed on micro-crack formation and their reduction using µ-PAW in dissimilar welding of Inconel 718 to AISI316L. They stated that, at high welding speeds, a columnar dendritic structure prevailed in the weld bead. Whereas, at low welding speeds, an equiaxed structure was found in the weld interior. Decreasing the welding speed can reduce the presence of deteriorating phases, such as Fe_2_Nb, NbC, and TiC intermetallics. Attempts were made to join AISI 316L and IN 718 using electrically assisted pressure joining by Li et al. [17]. They reported that a sound solid-state joint was fabricated for the dissimilar material combination based on microstructural and mechanical analysis, but further evaluation is needed for PWHT.

Anitha et al. [18] studied the influence of friction welding on the microstructure and mechanical properties of IN 718-SS410 weldments. They found that increasing rotational speed and friction pressure increased tensile strength, with the highest UTS of 718 MPa observed. The effect of rotational speed on microstructural representation at the interface was also investigated, and similar studies were reported by Anandaraj et al. [19]. They reported maximum UTS of 652 MPa at a speed of 1300 rpm and at a friction pressure of 220 MPa. Friction pressure plays a vital role in controlling mechanical properties. Murali et al. [20] investigated the microstructure characterization of friction-welded carbon steel and Inconel 718. They reported that austenite distribution in the deformed regions enriched in both directions. Longer widths of HAZ and TMAZ were observed on the carbon steel side, which eventually affected the microstructural features. The formation of the intermixing zone at the weld interface with fine grain structure was observed as a result of Fe, Ni, and Cr diffusion. The width of the intermixing zone varied from the centre to the periphery of the weldment [21].

Kong et al. [22] also investigated the effect of friction welding on microstructure and mechanical properties of IN718 in both the PWHT and as-welded conditions. They found that a rotational speed of 2000 rpm, friction pressure of 100–200 MPa, upset pressure of 100–200 MPa, and friction time of 10 s produced a fine-grained microstructure in the weld region of the friction-welded joints, with precipitates evenly distributed throughout the matrix. Tensile testing revealed that post-weld heat-treated samples exhibited a significant improvement in UTS (1485 MPa) compared to the as-welded samples (UTS of 950 MPa). However, the post-heat-treated joints showed lower % El values than the as-welded joints, indicating lower ductility. The presence of precipitates located at the grain boundaries of the weld zone could be the main reason for the improvement in strength after PWHT. Kimura et al. [23] investigated the joining of a nickel-based superalloy (Alloy 617) and heat-resistant steel (HRS) using friction welding. The joints were prepared with various welding parameter values, including a rotational speed of 1650 rpm, forging pressure of 30–360 MPa, forging time of 6 s, friction pressure of 30–90 MPa, and friction time of 0.04–40 s. They observed that more flash was observed on the HRS side compared to Alloy 617, which may be attributed to the drop in flow stress of the HRS at high temperatures. The authors concluded that a dissimilar joint could be created by using a precise duration of friction time and applying high forging pressure, specifically around 360 MPa. The microstructure and mechanical property correlation of dissimilar friction-welded joints (Inconel 751/austenitic steel 21–4N) were investigated by Zhu et al. [24]. The joints were prepared using inertia friction welding with a rotational speed of 5500 rpm, a maximum torque of 70 N.M, an upset pressure of 430 MPa, a welding time of 5 s, and a forging time of 2.5 s. Microstructure analysis revealed the presence of carbides in the Inconel 751 side of the joint and a chemical mixture zone at the interface (80 µm) between the two materials. The distribution of strength across the welds was found to be influenced by the carbide distribution and the chemical mixture zone.

Damodaram et al. [25,26] investigated the effect of PWHT on friction-welded IN 718. They reported that double aging treatment after welding improved mechanical properties. This is due to the generation of precipitates during PWHT. Kim et al. [27] explored the effect of PWHT on the mechanical properties of friction-welded Alloy 718 and SNCrW (in wt% 20 Cr, 10 Ni, 2 W, 1.4 Si, and 66.6 Fe) stainless steel. They found that PWHT samples resulted in good mechanical properties due to the formation of the γ″ phase. The tensile sample without PWHT failed in the weld joint. It was observed that the PWHT specimen fractured in the SNCrW parent metal due to the formation of precipitates. The peak temperature at the weld interface showed a significant effect on the microstructure and mechanical properties of the friction welded joints. Huang et al. [28] studied dissimilar friction welding of Alloy 720 Li to IN 718. They reported that the high γ′ volume fraction in Alloy 720 Li had a drop in hardness near the weld interface due to the precipitate dissolution. IN 718 has a similar soft zone beside the weld line due to delta δ phase dissolution, while the microhardness observed in this region is only half of the base material. The duration of PWHT aging impacts the coarsening of precipitates. Additionally, recrystallization occurred at the weld interface, resulting in fine grains on either side of the weld.

As mentioned earlier, the dissimilar joining of Inconel 718 with many austenitic and carbon steels is widely investigated. However, the combination of Inconel 718 and low-grade authentic stainless steel SS316L gives the right mix of properties for marine and high-temperature applications. To the author’s best knowledge, there are limited reports on dissimilar friction welding of AISI 316L to Inconel 718. The present study aimed to evaluate the effect of rotary friction welding parameters on tensile properties, hardness, macrostructure, and microstructure of dissimilar AISI 316L to IN 718 welds in the as-welded and post-weld direct aging conditions.

## 2. Experimental Procedure

The base metals used in the present study were IN 718 and AISI 316L rods with a diameter of 15 mm and a length of 100 mm. Table 1 shows the chemical compositions of the IN 718 and AISI 316L base metals. The IN 718 and AISI 316L rods were face-turned in a direction normal to the rotational axis and cleaned with acetone to degrease and remove any contaminants before friction welding. Inconel 718 underwent two different pre-weld heat treatments [29]. The first treatment involved a solution treatment (designated as ST). The second treatment involved a double aging heat treatment in addition to solution treatment (designated as STA). The pre-weld heat-treated IN 718 samples (ST and STA conditions) were friction welded with AISI 316L, designated as AISI 316L/IN 718 ST and AISI 316L/IN 718 STA, respectively. After friction welding, some AISI 316L/IN 718 ST samples underwent post-weld direct aging treatment, referred to as PWHT. Details of the heat treatment cycles are given in Table 2.

**Figure 1:**
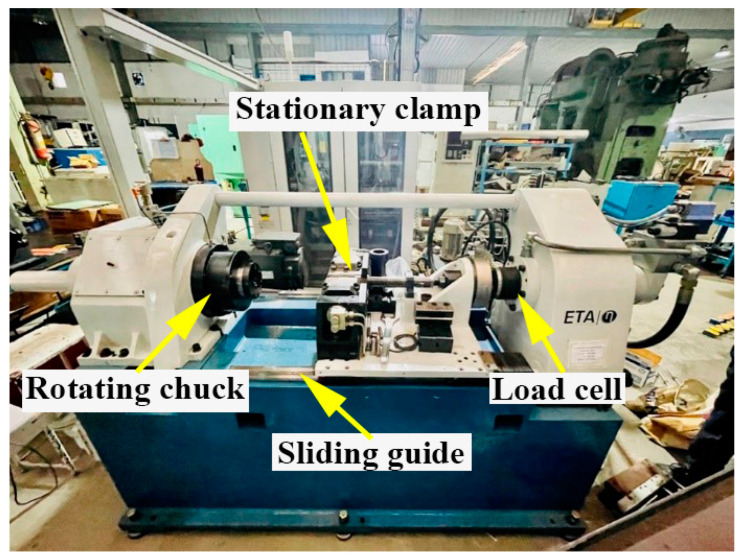
Rotary friction welding setup used to weld-dissimilar AISI 316L/IN 718 combinations.

A friction welding machine with a continuous drive (ETA Technology, Bangalore, India) and a maximum axial force (100 kN) was used in this study for preparing the welds. The friction welding parameters used are listed in Table 3. The welding technique for the rotary friction welding equipment was described in the reference [30]. In a standard friction welding cycle, five process parameters are utilized: spindle speed, forge pressure, forge time, friction time, and friction pressure. The first three parameters are from the friction stage of the weld cycle, while the last two are from the upset stage. The heat required to bond the parent metals was derived from the first stage, i.e., the friction stage. The bond between parent metals was then consolidated in the second stage, i.e., the upsetting/forging stage. The spindle speed, forge pressure, and forge time were all fixed at one set after initial testing. The friction pressure, forge pressure, and friction burn-off length were individually modified, while the remaining parameters were kept constant. Friction pressures below 190 MPa were deemed inadequate due to the extended duration of the friction stage. In addition, the flash seemed to be brittle, indicating insufficient heat generation to soften the parent metals properly. Various factors controlling the friction welding of IN 718 and steels were explained earlier [31,32]. By fixing the friction pressure at 197 MPa, the friction burn-off length was varied in 1 mm increments. At 4 mm, the flash exhibited an optimal appearance. Figure 1 shows the rotary friction welding setup used to weld dissimilar AISI 316L/IN 718 combinations.

For metallographic analysis, the dissimilar AISI 316L/IN 718 friction-welded samples were cut perpendicularly aligned to the weld and then polished. The AISI 316L side underwent a microstructural evaluation, utilizing an electrochemical etchant made up of a combination of 60 mL HNO_3_ and 40 mL deionized water (2 V DC, 10–15 s), whereas the IN 718 side was treated with 90 mL deionized water and 10 g oxalic acid (3 V DC, 10–15 s). A stereomicroscope (Nikon Instruments) was used to analyze the macrostructure, and an optical microscope (Motic Instruments) was used to study the microstructure. A scanning electron microscope equipped with EDS (Oxford Instruments) was used to conduct high-resolution microstructural analyses. The accelerating voltage of 15 kV was used during the SEM investigation. The line intercept method and ImageJ software were employed to determine the grain size. Vickers microhardness was measured across the weld zone using a pyramid indenter (Shimadzu Scientific Instruments) under a force of 200 g for 15 s. During the hardness tests, the indentations were 0.5 mm apart from the centre. According to ASTM E8 standard, the tensile specimens were extracted from the welds, and tensile tests were performed at a constant displacement rate of 1 mm/min on base metals, as well as friction welded specimens using a universal testing machine (BISS Instruments, Bangalore, India).

## 3. Results

### 3.1. Base Metal Microstructure

**Figure 2:**
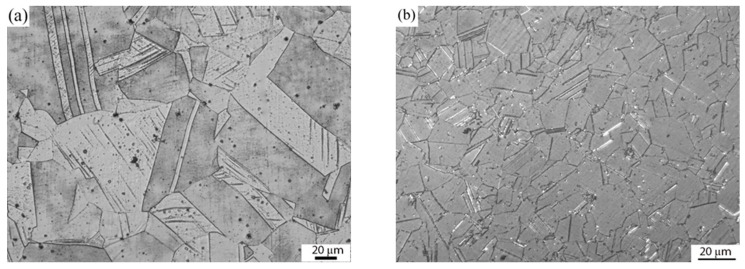

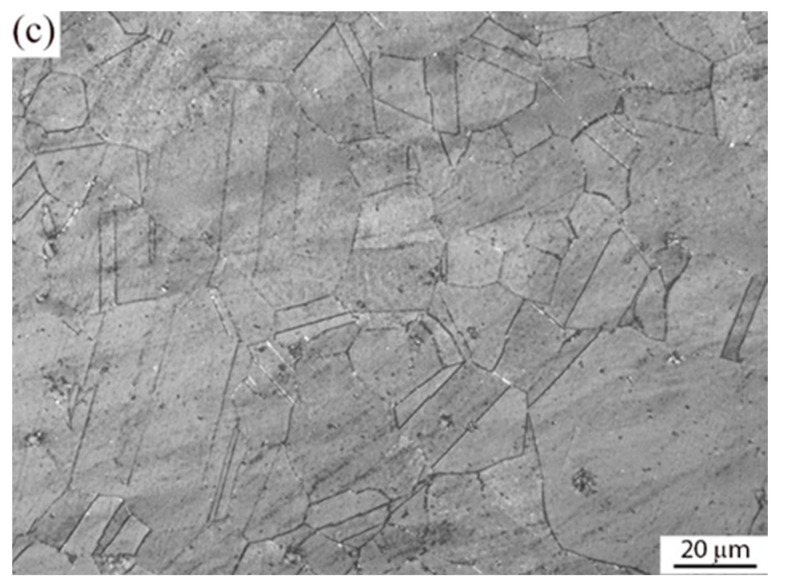
Optical micrographs of (**a**) AISI 316L, (**b**) Inconel 718 ST, and (**c**) Inconel 718 STA base metals.

**Figure 3:**
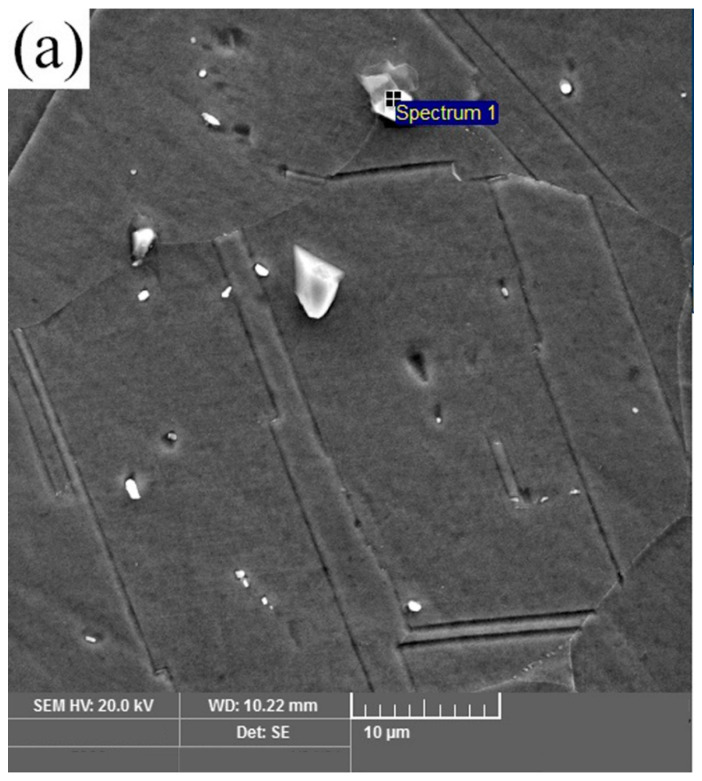

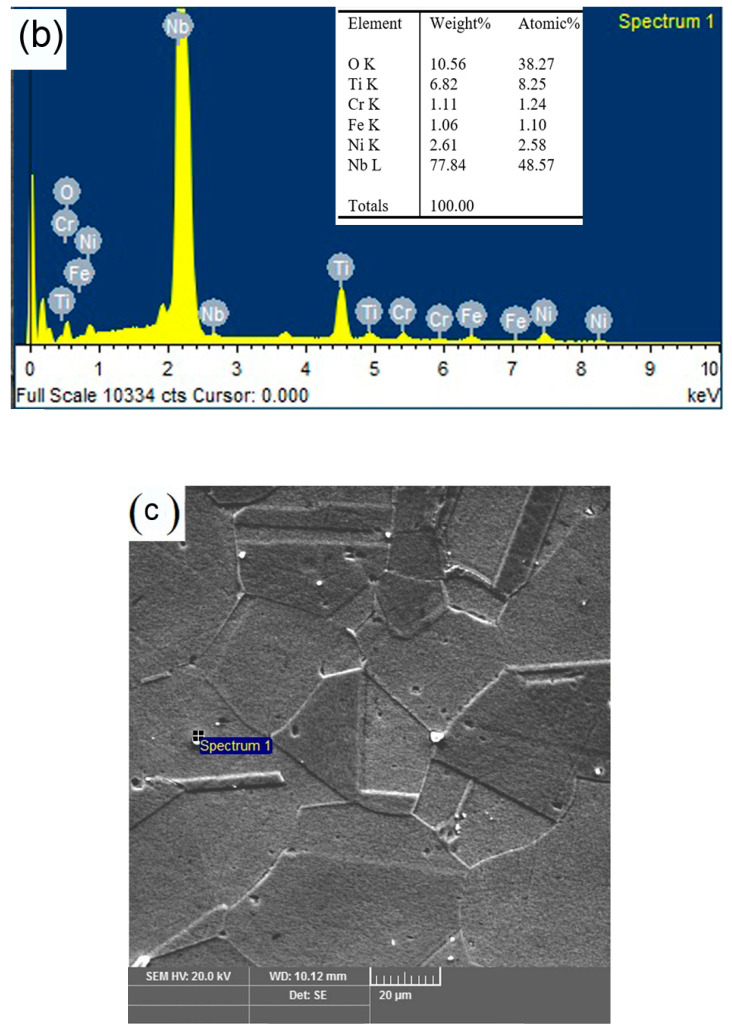

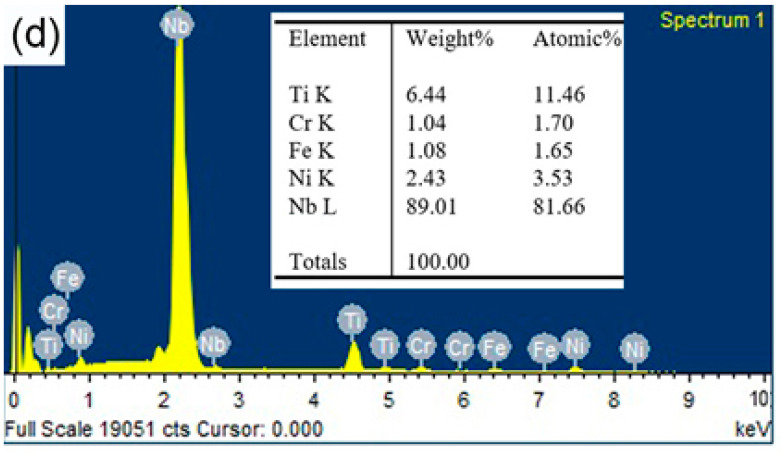
Scanning electron micrographs and EDS analysis of the precipitates in (**a**,**b**) Inconel 718 ST and (**c**,**d**) Inconel 718 STA base metals, respectively.

Figure 2a–c shows optical images of AISI 316L, IN 718 ST, and IN 718 STA, respectively. The optical image in Figure 2a shows an equiaxed fine-grain structure in AISI 316L with an average grain size of 63 ± 5 µm. Twins can be observed in the microstructure of AISI 316L. Inconel 718 base material displays an equiaxed grain structure in both ST and STA conditions (Figure 2b,c). Furthermore, the grain size of Inconel 718 in the STA condition is coarser than in the ST condition, with an average grain size of 23 ± 2 µm and 32 ± 3 µm, respectively. The STA sample is exposed to elevated temperatures for a more extended period during the aging treatment, resulting in grain coarsening. SEM and EDS analysis of the alloy IN 718 in ST and STA conditions are shown in Figure 3a–d, respectively. Figure 3a,c depict fine precipitates within the grain and grain boundaries. EDS analysis (Figure 3b,d) showed that the precipitates identified as γ″ are rich in Nb [25].

### 3.2. Macrostructure of Welds

**Figure 4:**
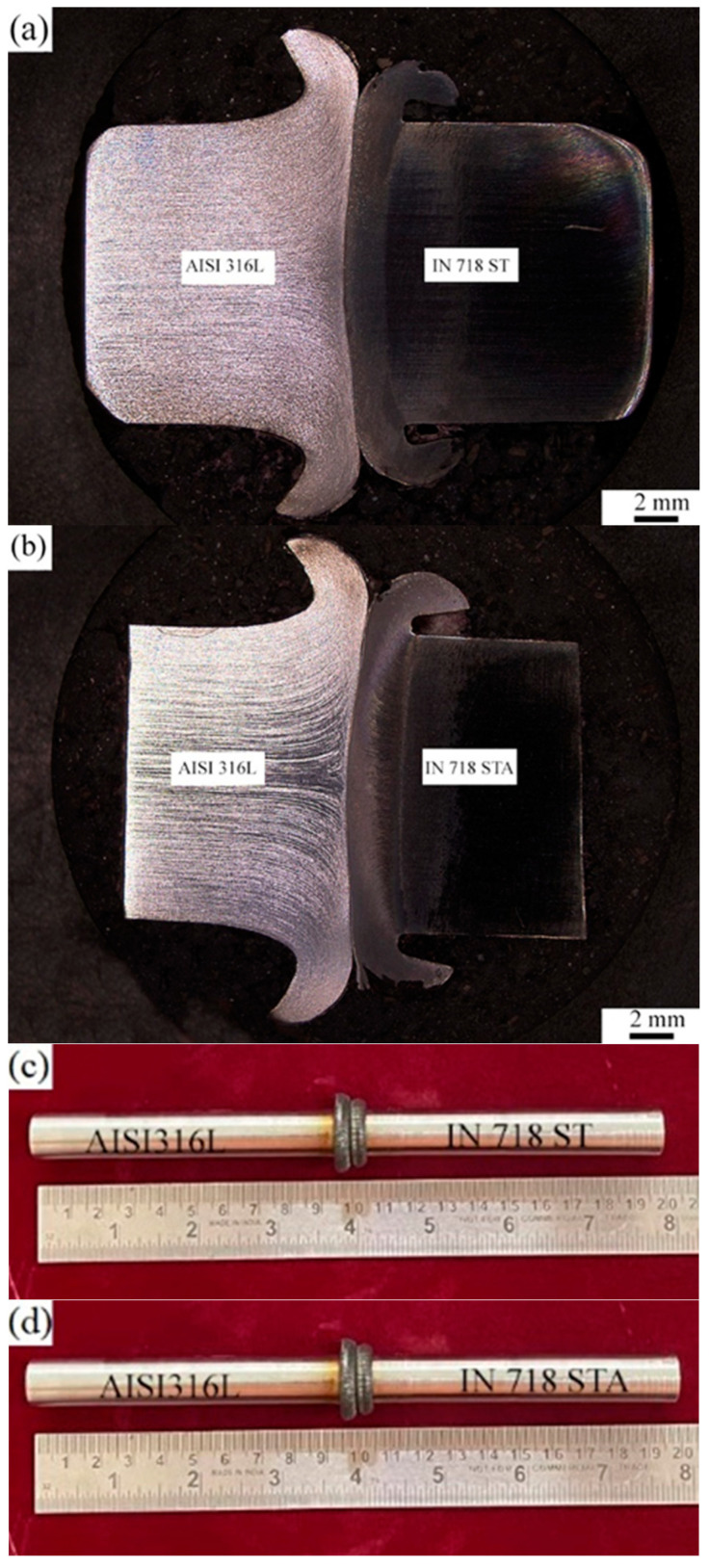
Macrograph and visual view of dissimilar friction welds AISI 316L/IN 718 ST (**a**,**c**) and AISI 316L/IN 718 STA (**b**,**d**).

The macrographs of the typical dissimilar friction-welded joints of AISI 316L/IN 718 ST and AISI 316L/IN 718 STA are shown in Figure 4a,b, respectively. The weld joints have good flash, suggesting that the heat generated by frictional forces is sufficient to plasticize the metals at the interface. The higher flash on the AISI 316L side and less flash on the IN 718 side may be due to the drop-in flow stresses of AISI 316L at elevated temperatures. The extent to which IN 718 and AISI 316L deform as a flash is dependent on their YS at high temperatures. As IN 718 has higher strength compared to AISI 316L, AISI 316L has more flash than IN 718. Native oxides and other impurities present on the base metal surface are removed in the form of plasticized material. The forge/upset pressure is high enough to extrude the plasticized material out of the weld joint, resulting in a sound weld joint without any cracks, as shown in the visual view of AISI 316L/IN 718 ST and AISI 316L/IN 718 STA in Figure 4c,d, respectively.

Friction welding has the advantage of requiring fewer requirements for welding surfaces. Impurities on the surfaces are forced away, ensuring the contact between surfaces is free from contaminants, resulting in a sound and defect-free weld. However, this idealistic situation fails when two base materials with distinct flow properties are combined. The absence of metal flow on one side, as indicated by the presence of material, impedes the desired cleaning action. However, in the present work, there was some deformations observed in Inconel 718 side (Figure 4a,b), somewhat alleviating the self-cleaning problem, resulting in a weld joint free of delaminated regions and welding defects.

### 3.3. Microstructure of Welds

**Figure 5:**
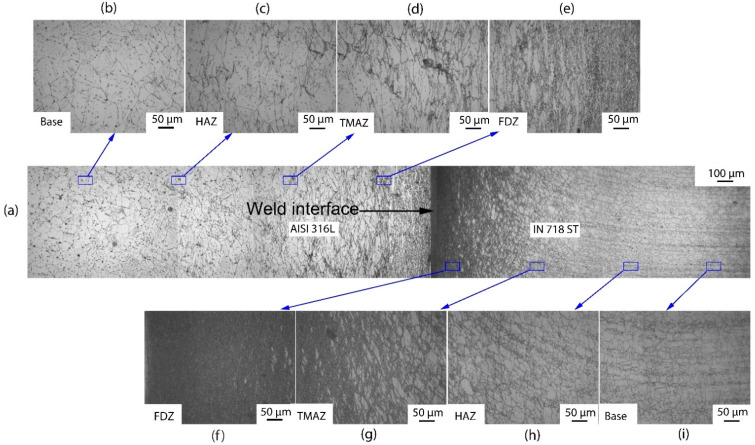
Cross-sectional view of weld joint interface of dissimilar friction weld AISI 316L-IN718 ST condition (**a**) weld interface; (**b**) base metal on AISI 316L side; (**c**) HAZ on AISI 316L side; (**d**) TMAZ on AISI 316L side; (**e**) FDZ on AISI 316L side; (**f**) FDZ on IN718 side; (**g**) TMAZ on IN718 side; (**h**) HAZ on IN718 side; and (**i**) base metal on IN718 side.

**Figure 6:**
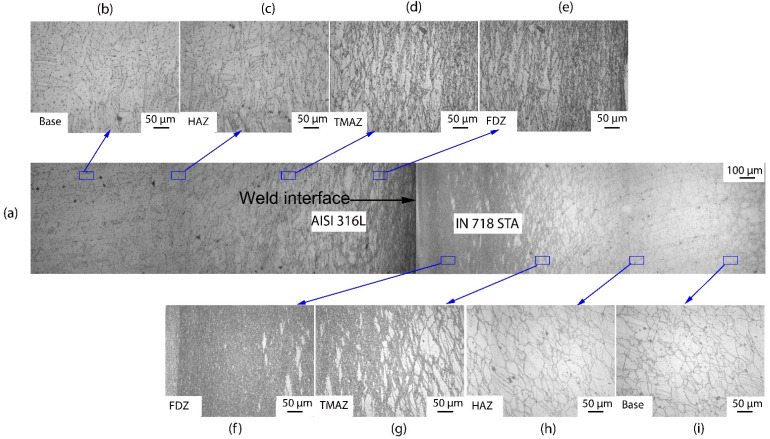
Cross-sectional view of weld joint interface of dissimilar friction weld AISI 316L-IN718 STA condition: (**a**) weld interface; (**b**) base metal on AISI 316L side; (**c**) HAZ on AISI 316L side; (**d**) TMAZ on AISI 316L side; (**e**) FDZ on AISI 316L side; (**f**) FDZ on IN718 side; (**g**) TMAZ on IN718 side; (**h**) HAZ on IN718 side; and (**i**) base metal on IN718 side.

**Figure 7:**
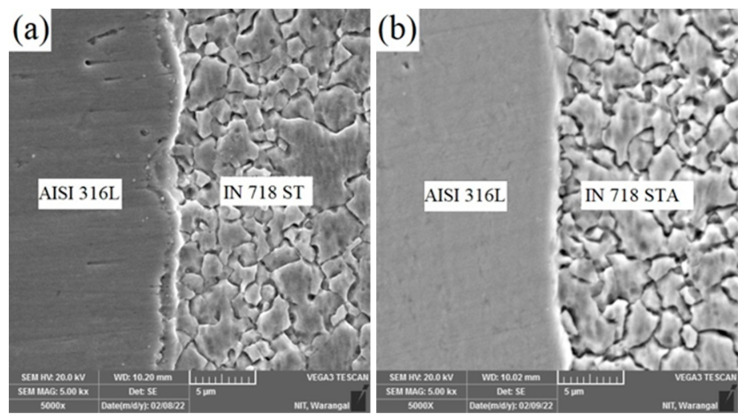
Scanning electron micrograph of dissimilar friction welds (**a**) AISI316L/IN718 ST and (**b**) AISI316/-IN718 STA.

The optical microscopic images of the weld cross-section (Figure 5 and Figure 6) of dissimilar friction welded specimens AISI 316L/IN 718 ST and AISI 316L/IN 718 STA, respectively, show distinctive regions: FDZ, TMAZ, HAZ, and BM on either side of the weld interface. In both conditions, FDZ reveals an entirely recrystallized fine-grained structure. The average grain size of FDZ for AISI 316L/IN 718 ST is 5 ± 2 µm on the Inconel side (Figure 5f) and 6 ± 2 µm on the AISI 316L side (Figure 5e). In AISI 316L/IN 718 STA condition, it is observed to be 8 ± 2 µm on the Inconel side (Figure 6f) and 7 ± 3 µm on the AISI 316L side (Figure 5e). The severe plastic deformation during friction welding increases the dislocation density of defects, which imparts strain energy into the material. Recrystallization is a process dependent on parameters, such as prior grain size, chemical composition, etc., where the system’s energy becomes reduced [33]. Dynamic recrystallization, on the other hand, is a process in which very fine grains are formed in thermomechanically treated materials. Continuous dynamic recrystallization is commonly observed in metals, such as Inconel, whose stacking fault energy is high when heated to temperatures above 50% of their melting point [34]. The material is subjected to substantial plastic deformation during the friction welding process. During plastic deformation, the grains become fractured, and as a result, several low-angle grain boundaries with misorientation develop and act as highly favourable sites for the nucleation of fine recrystallized grains. The formed nuclei grow, consume the deformed grains, and lead to the development of a fine equiaxed microstructure. Although the materials in rotary friction welding do not reach their melting point during the welding process, the temperature at the weld interface can reach 0.7–0.8 times the melting point, resulting in the formation of new nucleation sites and a reduction in grain size [35].

A notable change in terms of elongated grain boundaries and grain size is observed between the FDZ and the TMAZ. As TMAZ is subjected to less heat and less plastic deformation than the FDZ, elongated grains are observed in this zone. In addition, coarse grains are found in TMAZ. Incomplete recrystallization occurs in the TMAZ due to lower heat input and plastic deformation. Consequently, bimodal grains are present in the TMAZ, consisting of both finer recrystallized grains and coarser grains that have not undergone recrystallization. In the case of AISI 316L/IN 718 ST, the average grain size in the TMAZ is 17 ± 5 µm on the Inconel side (Figure 5g) and 30 ± 9 µm on the AISI 316L side (Figure 5d). In contrast, the AISI 316L/IN 718 STA joint is observed to be 23 ± 6 µm on the Inconel side (Figure 6g) and 32 ± 7 µm on the AISI 316L side (Figure 5d). In the HAZ of the welded joint, grain coarsening is observed. As HAZ experiences higher temperatures and plastic deformation, the size of grains in HAZ is coarser than that of the BM. The average grain size of HAZ in the AISI 316L/IN 718 ST condition is observed to be 36 ± 7 µm on the Inconel side (Figure 5h) and 69 ± 5 µm on the AISI 316L side (Figure 5c). Whereas, in the AISI 316L/IN 718 STA condition, it is observed to be 40 ± 9 µm on the Inconel side (Figure 6h) and 73 ± 6 µm on the AISI 316L side (Figure 6c). The unaffected base metal has a grain size of 26 ± 4 µm and 34 ± 4 µm on the Inconel side in AISI 316L/IN 718 ST, AISI 316L/IN 718 STA (Figure 5i and Figure 6i), respectively. In contrast, it is 64 µm for the AISI 316L side in both conditions AISI 316L/IN 718 ST, AISI 316L/IN 718 STA (Figure 5b and Figure 6b respectively).

In the FDZ, the average grain size is very small due to recrystallization, regardless of the material condition. In the TMAZ, a bimodal grain structure is observed, with a larger grain size than the FDZ on both Inconel and 316L sides. Similar to the FDZ zone, the material condition has a slight effect on grain size in the TMAZ. In the HAZ, the grain size is the largest among all the zones for both Inconel and 316L, and the material condition has shown an effect on grain size in the HAZ for both materials. Table 4 outlines the grain sizes of the various zones (as-welded and PWHT) conditions.

SEM micrographs of dissimilar weldments AISI 316L/IN 718 ST and AISI 316L/IN 718 STA at the weld interface are shown in Figure 7a,b, respectively. The AISI 316L side does not have any precipitates, irrespective of the weld condition. In the FDZ on the Inconel side, the precipitates are completely dissolved due to the high temperature, and reprecipitation does not occur due to the lack of time.

**Figure 8:**
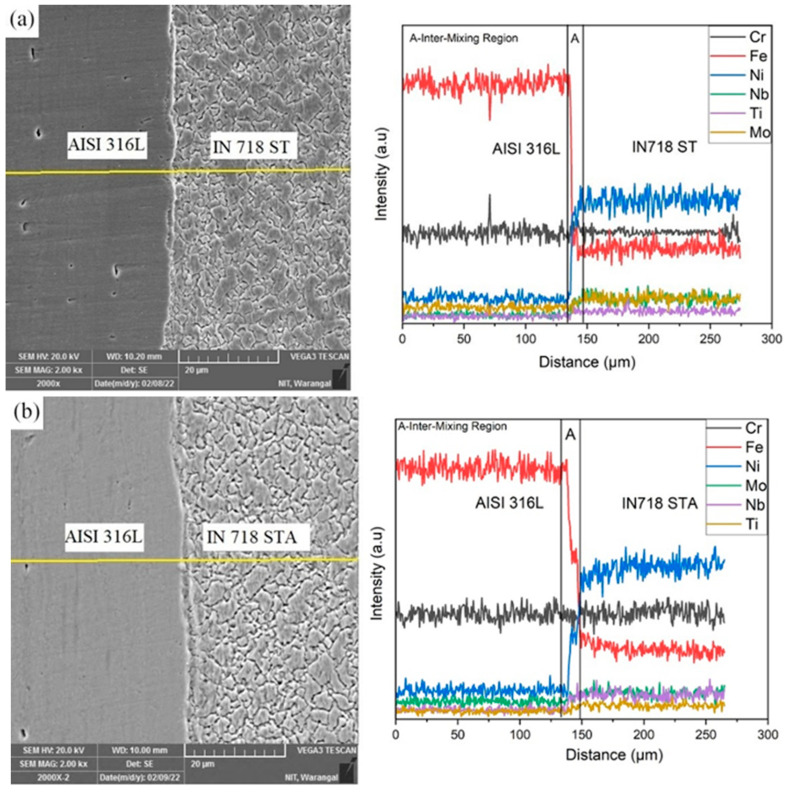
SEM micrograph of dissimilar friction welds (**a**) AISI316L-IN718 ST with EDS line scan and (**b**) AISI316L-IN718 STA with EDS line scan.

The compositional variation across the weld interfaces has been investigated using line scan data. Figure 8a,b shows the EDS line scan analysis of the dissimilar AISI 316L/IN 718 ST and AISI 316L/IN 718 STA interfaces, respectively. During friction welding, when higher temperatures are present, and the materials are extensively plasticized, mechanical intermixing and diffusion are more likely to occur across the interface. The SEM micrograph shows the intermixed zone of the IN 718 and AISI 316L. An intermixing zone of IN 718 and AISI 316L is generated at the weld interface as a result of the material softening, extruding, and squeezing at higher rotating speeds during friction welding. At the weld interface, a variation in composition is observed, as depicted in Figure 8a,b. A small amount of Fe has diffused from the AISI 316L to the Inconel 718 side, and the elements Ni, Nb, and Ti have diffused from IN 718 to the AISI 316L side [12].

**Figure 9:**
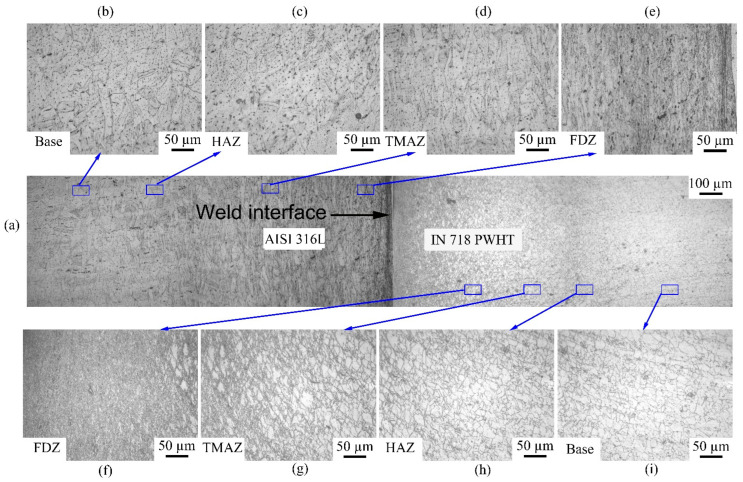
Cross-sectional view of weld joint interface of dissimilar friction weld AISI 316L-IN718 PWHT condition (**a**) weld interface; (**b**) base metal on AISI 316L side; (**c**) HAZ on AISI 316L side; (**d**) TMAZ on AISI 316L side; (**e**) FDZ on AISI 316L side; (**f**) FDZ on IN718 side; (**g**) TMAZ on IN718 side; (**h**) HAZ on IN718 side; and (**i**) base metal on IN718 side.

Figure 9 shows a micrograph of different zones of the AISI 316L/IN 718 PWHT dissimilar friction welded samples. The zones in PWHT samples were similar compared to other conditions. The grain size on AISI 316L side had slightly increased as compared to as-welded conditions due to prolonged time in elevated temperature during post-weld heat treatment. However, grain size variation between zones is similar to as-welded conditions. The average grain size on the Inconel side in FDZ, TMAZ, HAZ, and base regions are 9 ± 3 µm, 25 ± 8 µm, 42 ± 6 µm, and 36 ± 5 µm, respectively, and 9 ± 3 µm, 35 ± 6 µm, 76 ± 6 µm, and 69 ± 6 µm, respectively, on the AISI 316L side.

### 3.4. Mechanical Properties

**Figure 10:**
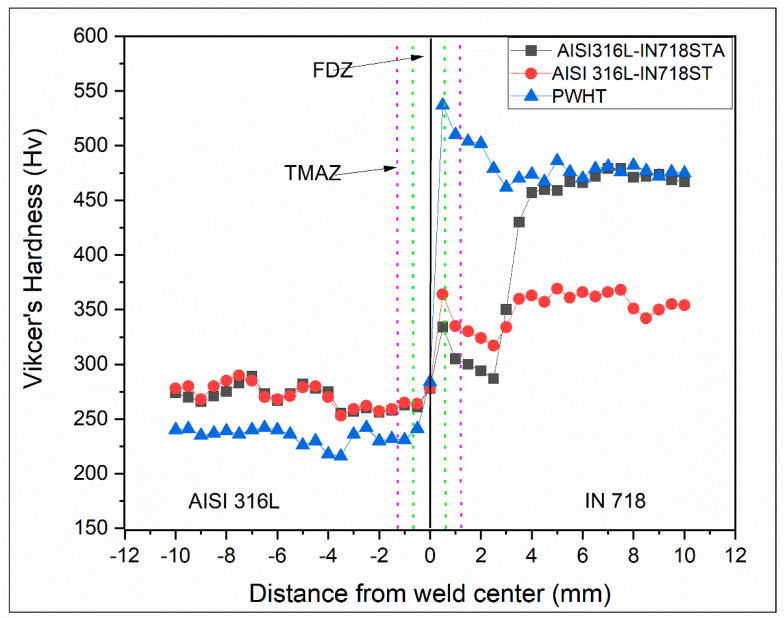
The microhardness distribution across the weld interface of dissimilar friction welds AISI316L-IN718 ST, AISI316L-IN718 STA, and PWHT.

Figure 10 shows the Vickers microhardness profiles across the interface of dissimilar weldments AISI 316/IN 718 ST, AISI 316L/IN 718 STA in the as-welded and AISI 316L/IN 718 ST PWHT conditions. In the FDZ regions on the IN 718 side, it is observed that there is a decrease in hardness on AISI 316L/IN 718 STA compared with AISI 316L/IN 718 ST. The loss in hardness on the Inconel 718 side of the AISI 316L/IN 718 STA sample could be due to the dissolution of strengthening precipitates, which could be attributed to the extremely high temperatures reached during the friction welding process. During welding, temperatures as high as 1100 °C have been reported at the weld contact of a friction weld, which is hot enough to dissolve the strengthening precipitates. Cooling rates are insufficient to re-precipitate, and the hardness loss cannot be reversed unless a post-weld heat treatment is performed. High hardness is observed in FDZ on the Inconel 718 side of the AISI 316L/IN 718 ST sample when compared to AISI 316L/IN 718 STA. This may be attributed to the strain hardening of the FDZ of Inconel 718 in AISI 316L/IN 718 ST, which is higher compared to the AISI 316L/IN 718 STA condition. IN 718 ST softened at a temperature lower than IN 718 STA, resulting in severe plastic deformation [36].

Due to the peak temperatures and high strain energy encountered by the FDZ of IN 718, it exhibited higher recrystallization in the ST condition compared to the STA condition. This can be correlated with the finer grain size obtained in the ST condition compared to the STA condition, and thus the FDZ of IN 718 ST has higher hardness, comparatively. It is reported that the PWHTed sample exhibited the highest hardness on the weld interface of the Inconel 718 side due to the precipitation of strengthening γʹ and γ″ precipitates. However, the TMAZ and HAZ have lower hardness values compared to the FDZ in all the conditions on either side due to an increase in grain size as moving away from the weld interface. An increase in hardness of the unaffected base region in AISI 316L/IN 718 ST, AISI 316L/IN 718 STA, and PWHT compared to the TMAZ and HAZ regions due to the availability of precipitates hinders the dislocation motion and grain size effect also observed (see Table 4). The FDZ and TMAZ regions on the AISI 316L side of the as-welded conditions showed similar hardness values, as AISI 316L is not involved in any heat treatment process before welding. In contrast, the sample showed the lowest hardness values on the AISI 316L side of the PWHT condition. Continuous exposure to high temperatures during the aging treatment might be linked to grain growth and stress relief. An average hardness of 260 HV is obtained in the as-received AISI 316L base material, and 375 HV and 470 HV values, on average, are shown in Inconel 718 ST and Inconel 718 STA base metals, respectively.

**Figure 11:**
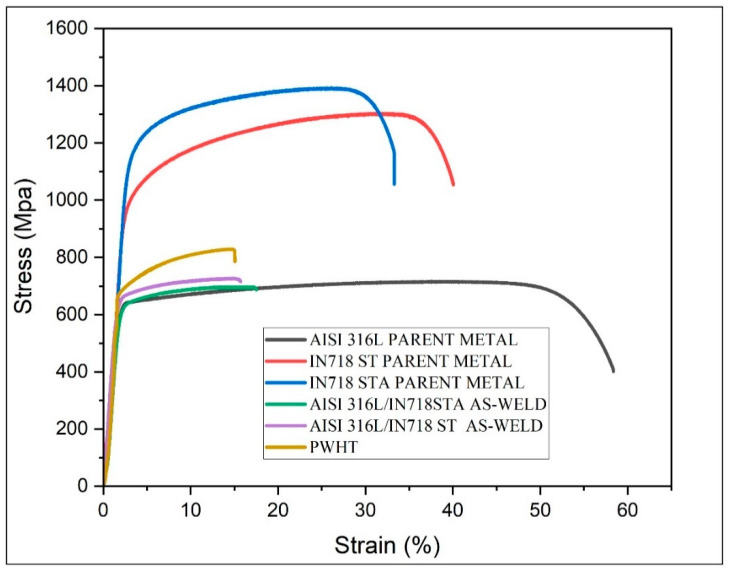
Typical tensile curves of parent metals and friction welded joints.

The tensile properties of the dissimilar weldments, including AISI 316L/IN 718 ST and AISI 316L/IN 718 STA, in both as-welded and AISI 316L/IN 718 ST PWHT conditions, were evaluated through tensile tests. The corresponding tensile curves are presented in Figure 11. From the stress vs. strain curves, the following points are noted. The AISI 316L/IN 718 ST welds showed a YS and UTS of 634 ± 9 MPa and 728 ± 7 MPa, respectively, while the % El was about 14 ± 1.5%. In contrast, the AISI 316L/IN 718 STA weldment showed YS and UTS of 602 ± 3 MPa and 697 ± 2 MPa, respectively, while the % El was about 17 ± 0.9%. As previously stated, the effect of dynamic recrystallization during the welding process in AISI 316L/IN 718 ST was higher than in AISI 316L/IN 718 STA, resulting in a drop in strength values for the latter condition. Compared to the other welds (PWHT, ST and STA), the AISI 316L/IN718 PWHT welds demonstrated greater strength (YS: 730 ± 2 MPa, UTS: 828 ± 5 MPa) but lower ductility (%El: 9 ± 1.2) (Table 5). The reason for this is likely that AISI 316L is not heat-treatable, and therefore, the PWHT only increased the strength of IN718. The increase in strength observed in the post-weld heat-treated sample of the IN718 side is likely due to the formation of strengthening precipitates, such as γ′ and γ″. This corresponds with the findings of Lalam et al. [11], and they also noted similar results in their investigation of dissimilar metal combinations involving IN718 and EN24 friction welding. The stress vs strain plots of the base metal are also included in Figure 11 for comparison. The strength of the weldments was higher than that of 316L BM and lower than that of IN 718 BM, irrespective of the material condition. However, the weldments exhibited lower ductility compared to both base metals. Dissolution of precipitates during the welding process resulted in lower strength as compared to the Inconel base materials. The tensile properties, such as YS, UTS, and % El, are mentioned in Table 5.

**Figure 12:**
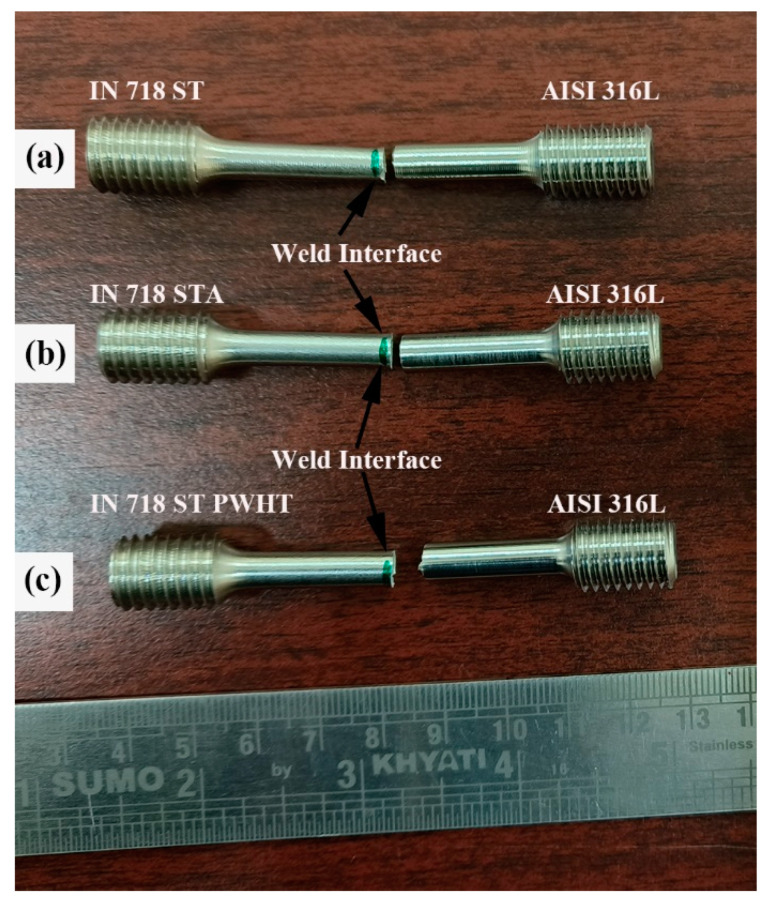
Tensile failure location of the dissimilar friction welds (**a**) AISI 316L-IN718 ST and (**b**) AISI 316L-IN718 STA and (**c**) PWHT.

**Figure 13:**
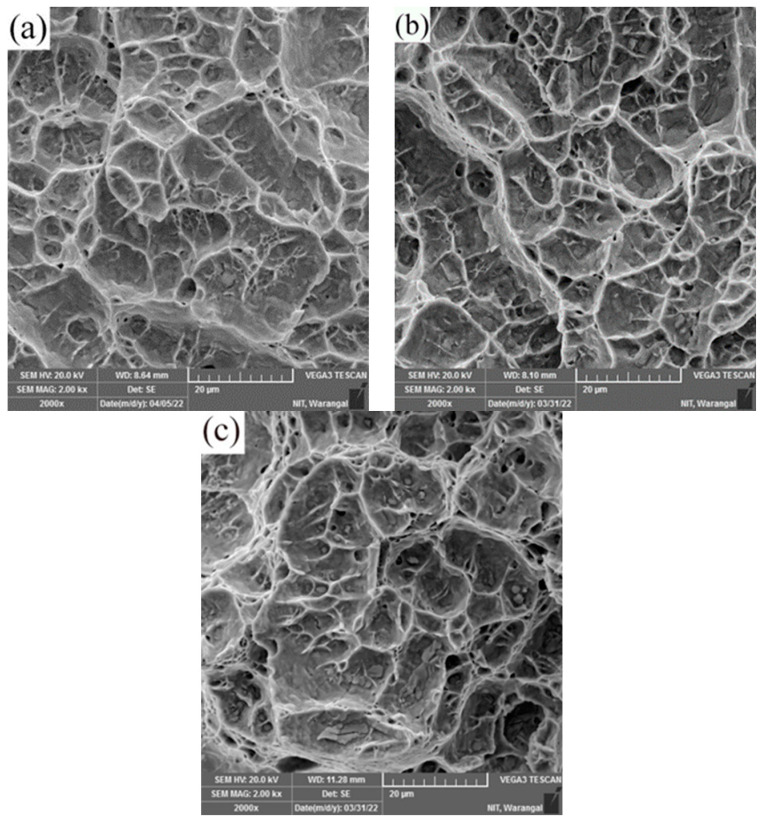
Tensile fracture morphology of dissimilar friction welds (**a**) AISI316L/IN718 ST, (**b**) AISI316/-IN718 STA, and (**c**) PWHT.

The dissimilar welds AISI 316L/IN 718 ST and AISI 316L/IN 718 STA in both as-welded and PWHT conditions have broken in the HAZ region on the AISI 316L side. The failure of the samples in the HAZ region of AISI 316L indicates that the joint produced is sound and strong. The images indicating the failure location of the weldments after the tensile test are shown in Figure 12. The specimen’s failure has been noted to occur not at its centre, but rather at a slight offset to the weld interface, specifically in the HAZ. In all conditions, the HAZ region’s grain size is larger compared to FDZ and TMAZ, and dissolution of precipitates is also observed in the region due to exposure to high temperature during welding, which could be attributed to the weldments’ failure. The secondary electron micrographs of dissimilar AISI 316L/IN 718 ST, AISI 316L/IN 718 STA, and PWHT weld joints’ tensile fractured surfaces are shown in Figure 13a–c, respectively.

On the fracture surface, fibrous characteristics with dimples are detected. The dimple fracture traits signify a ductile fracture mode, brought about by the initiation and creation of microvoids that ultimately merged to form bigger voids. This led to a ductile fracture when the tensile specimen’s remaining cross-sectional area was insufficient to hold the applied load. The PWHT weldments have coarser dimples, while the as-welded weldments have finer dimples.

## 4. Conclusions

In this study, the dissimilar AISI 316L/IN 718 ST and AISI 316L/IN 718 STA continuous friction welded joints were prepared, and PWHT was also performed on AISI 316L/IN 718 ST as-welded samples. The effect of post-weld aging treatment on microstructure and mechanical properties was studied. The following conclusions were drawn:

1. A defect-free, sound-dissimilar AISI 316L/IN 718 weld was obtained without any cracks or incomplete bonding in all conditions using the continuous drive friction welding process.
2. The macrostructure of the dissimilar AISI 316L/IN 718 weld illustrated that AISI 316L has more flash than IN 718, indicating that AISI 316L underwent more plastic deformation than IN 718 during the welding process.
3. The microstructure of the friction welds in AISI 316L/IN 718 ST and AISI 316L/IN 718 STA conditions revealed the dissolution of precipitates during welding. Recrystallization of grains resulted in a fine grain structure at the weld interface, where the peak temperature occurs during welding.
4. The dissimilar PWHT continuous friction welded sample resulted in the highest hardness among all conditions in the FDZ and TMAZ zone due to the formation of precipitates. However, on the AISI 316L side, there was a drop in hardness due to larger exposure to high temperatures, resulting in grain growth.
5. Tensile samples failed in the HAZ region for all dissimilar friction weld joints of AISI 316L/IN 718 ST, AISI 316L/IN 718 STA, and PWHT. The reason for this is that the HAZ has a larger grain size in comparison to both the weld interface and the base metal.
6. The dissimilar AISI 316L/IN 718 ST and AISI 316L/IN 718 STA friction weldments showed a slightly lower UTS and higher ductility (YS = 634 ± 9 MPa, UTS = 728 ± 7 MPa, 14 ± 1.5 % El and YS = 602 ± 3 MPa, UTS = 697 ± 2 MPa, 17 ± 0.9 % El, respectively) compared to the dissimilar weldment in PWHT condition (YS = 730 ± 2 MPa, UTS = 828 ± 5 MPa, 9 ± 1.2 % El). This may be attributed to the precipitate formation during PWHT and the dissolution of precipitates during the welding process in the STA condition.

## Figures and Tables

**Table 1:** Composition of base metals (wt %).

Elements	Fe	Cr	Mn	Ni	Mo	C	Si	P	S
AISI 316L	Bal	16.5	1.0	10.4	2.1	0.03	0.4	0.02	0.001
Elements	Fe	Cr	Ni	Mo	Nb	V	Ti	Al	Si
IN718	19.7	18.1	51.6	3.2	5.1	0.3	1.1	0.5	0.3

**Table 2:** Heat treatment details for Inconel 718.

Condition	Heat Treatment
ST condition- solution treatment	Holding at 980 °C/1 h
STA condition- solution treatment and aging	980 °C/1 h, air cooling to room temperature + 720 °C/8 h, furnace cooling + 620 °C/9 h, air cooling.
PWHT-ST post welded sample direct aging	720 °C/8 h, furnace cooling + 620 °C/9 h, air cooling

**Table 3:** Welding Parameters.

Parameter	AISI 316L/IN718 ST	AISI 316L/IN718 STA
Friction Pressure (MPa)	197	197
Forge Pressure (MPa)	394	394
Friction Burn-Off (mm)	4	4
Forge Time (s)	4	4
Total Burn-Off (mm)	8.465	8.26

**Table 4:** The average grain sizes of dissimilar friction weldments AISI 316L/IN718 ST, AISI 316L/IN718 STA, and PWHT.

Material	AISI 316L Side (µm)				IN 718 Side (µm)			
Condition	FDZ	TMAZ	HAZ	Base	FDZ	TMAZ	HAZ	Base
AISI 316L/IN718 ST	6 ± 2	30 ± 9	69 ± 5	64 ± 7	5 ± 2	17 ± 5	36 ± 7	26 ± 4
AISI 316L/IN718 STA	7 ± 3	32 ± 7	73 ± 6	64 ± 5	8 ± 2	23 ± 6	40 ± 9	34 ± 3
PWHT	9 ± 3	35 ± 6	76 ± 6	69 ± 3	9 ± 3	25 ± 8	42 ± 6	36 ± 5

**Table 5:** Results of tensile testing of dissimilar friction welds AISI316L/IN718ST, AISI316/IN718STA, PWHT, and parent materials.

Condition	Yield Strength (YS), MPa	Ultimate Tensile Strength (UTS), MPa	% Elongation (% El)
AISI316L/IN718 STA	602 ± 3	697 ± 2	17 ± 0.9
AISI316/IN718 ST	634 ± 9	728 ± 7	14 ± 1.5
PWHT	730 ± 2	828 ± 5	9 ± 1.2
IN718 ST Parent Material	999 ± 3	1304 ± 6	40 ± 2.9
IN718 STA Parent Material	1161 ± 5	1393 ± 4	33 ± 2
AISI 316L Parent Material	558 ± 2	716 ± 3	58 ± 3.2

## Data Availability

Data are contained within this article.
